# Acute bullous iododerma with cryptococcoid neutrophilic dermatosis: A case series

**DOI:** 10.1016/j.jdcr.2025.12.022

**Published:** 2026-01-30

**Authors:** Emily L. Chen, Yiwen Helen Li, Maya Collins, Justin T. Cheeley

**Affiliations:** a Department of Dermatology, Emory University School of Medicine, Atlanta, Georgia; b Department of General Internal Medicine, Emory University School of Medicine, Atlanta, Georgia

**Keywords:** antineutrophil cytoplasmic antibody (ANCA), bullous, chronic kidney disease, cryptococcoid neutrophilic dermatosis, end-stage renal disease, hydralazine, iodine, iododerma, radiocontrast, Sweet syndrome, vasculitis

## Introduction

Iododerma is a rare and diagnostically challenging eruption caused by iodine-containing compounds, often presenting with polymorphic features that clinically or histologically mimic Sweet syndrome, vasculitis, or autoimmune blistering dermatoses. We describe a case series of 3 patients with acute bullous iododerma secondary to antecedent radiocontrast exposure with histopathologic cryptococcoid neutrophilic dermatosis (CND).

## Case 1

**Fig 1:**
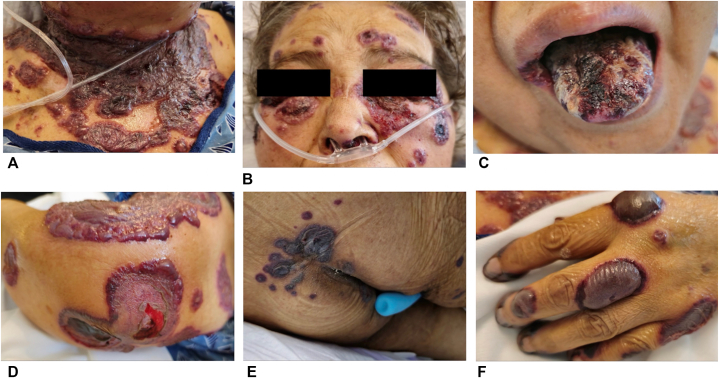
Acute bullous iododerma: violaceous, tense hemorrhagic, and serous-filled bullae in various stages on the **(A)** neck and proximal chest, **(B)** periorbital face, **(C)** tongue, **(D)** upper forearm, **(E)** gluteal region, and **(F)** left hand.

A woman in her fifties with hypothyroidism, hypertension, type 2 diabetes mellitus, heart failure with preserved ejection fraction, and end-stage renal disease receiving intermittent emergency department-based hemodialysis was transferred from an outside hospital for hypoxic respiratory failure and a bullous skin eruption. The eruption developed about 2-3 days after a computed tomography (CT) scan with intravenous (IV) contrast was performed to rule out pulmonary embolism. She developed tender, tense, erythemato-violaceous, and hemorrhagic bullae involving the face, oral cavity, scalp, upper torso, fingers, and perineum (Fig 1). A similar episode occurred 6 months earlier following IV contrast exposure, which resolved with corticosteroid therapy.

**Fig 2:**
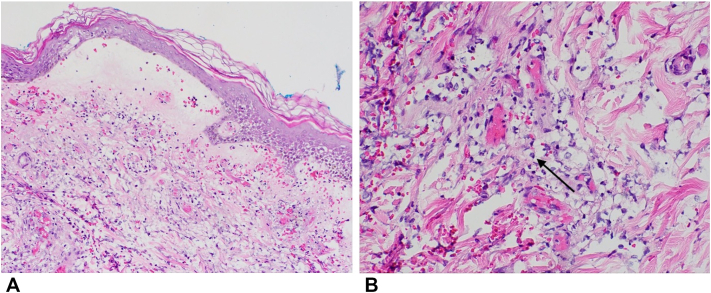
Acute bullous iododerma: **A,** subepidermal bulla with marked epidermal spongiosis and an associated mixed inflammatory infiltrate, 10× magnification; **B,** scant cryptococcoid bodies (*arrow*), 40× magnification. Hematoxylin and eosin stain.

Upon transfer to our hospital, her condition worsened following additional contrast-enhanced imaging. She was started on IV methylprednisolone (1 mg/kg), oral dapsone (50 mg daily), and triamcinolone ointment. Laboratory evaluation revealed a positive perinuclear antineutrophil cytoplasmic antibody (p-ANCA) titer >1:1280 (normal < 1:20), positive antimyeloperoxidase enzyme-linked immunosorbent assay at 70 (normal < 19 AU/mL), positive antinuclear antibodies, low C3 and C4, elevated rheumatoid factor, antihistone antibodies, and dysmorphic red blood cells on urine microscopy. Skin biopsy demonstrated a subepidermal bulla with marked epidermal spongiosis and an associated mixed inflammatory infiltrate containing scant cryptococcoid bodies (Fig 2). Direct and indirect immunofluorescence were nonspecific, and enzyme-linked immunosorbent assay for anti-BP180, anti-BP230, and anti–type VII collagen were negative. The differential diagnosis included hydralazine-induced ANCA vasculitis, Sweet syndrome, and hydralazine-induced lupus. Hydralazine was discontinued.

Despite initial therapy and regular hemodialysis sessions since admission, her condition worsened with continued bullae formation. High-dose IV corticosteroids and rituximab were administered, resulting in the cessation of new lesions and quiescence of old lesions. However, her clinical course was complicated by sepsis, and she was ultimately transitioned to hospice care and passed away.

## Case 2

**Fig 3:**
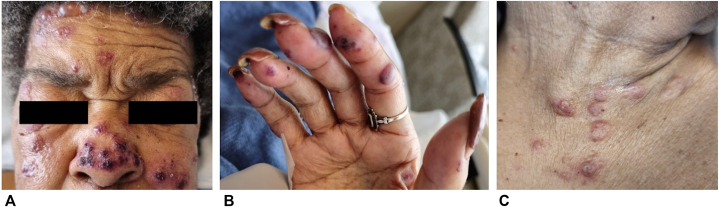
Acute bullous iododerma: several *red*, edematous vesicles and bullae, some with hemorrhagic crusting on **(A)** the face, **(B)** right hand, and **(C)** the neck and superior chest.

A woman in her seventies with heart failure with preserved ejection fraction, hypertension, type 2 diabetes mellitus, stage 3B chronic kidney disease, chronic obstructive pulmonary disease, and vascular dementia presented with a heart failure exacerbation. On hospital day 5, she developed sudden onset painful vesicobullae on the face, tongue, and bilateral upper extremities (Fig 3). Ten days prior, she had presented to another hospital after a fall where she underwent a CT abdomen and pelvis with IV iodinated contrast, as well as a non-contrast CT of the head and neck.

**Fig 4:**
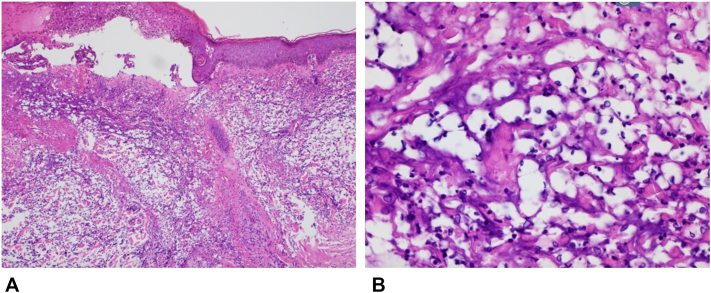
Acute bullous iododerma: dense dermal neutrophilic inflammatory infiltrate and vacuolated spaces with cryptococcoid bodies, **(A)** 10× and **(B)** 40× magnification. Hematoxylin and eosin stain.

A lesional punch biopsy from the left forearm revealed a dense neutrophilic dermal infiltrate with yeast-like bodies concerning for CND (Fig 4). Serum cryptococcal antigen and infectious workup were negative. Additional testing showed a positive p-ANCA titer >1:1280 (normal < 1:20) and negative direct immunofluorescence. Serum iodine levels obtained 1 day after symptom onset were markedly elevated at 17,991.9 μg/L (normal 40.0-92.0 μg/L). Given the recent iodinated contrast exposure, absence of fever, and histopathologic findings, acute iododerma was favored. She was treated with IV methylprednisolone and topical corticosteroids, resulting in significant improvement over the following week.

## Case 3

**Fig 5:**
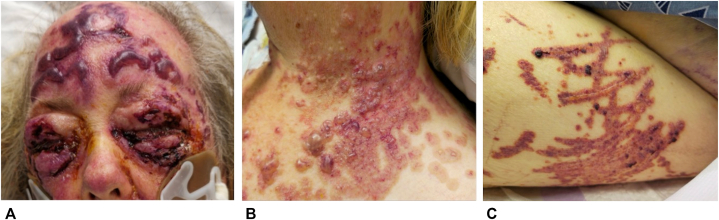
Acute bullous iododerma: *clustered large purple*, tense bullae with background purpura on **(A)** the face and forehead, **(B)** the left neck, and linear erythematous papules and plaques on **(C)** the left outer thigh at sites of patient excoriation.

**Fig 6:**
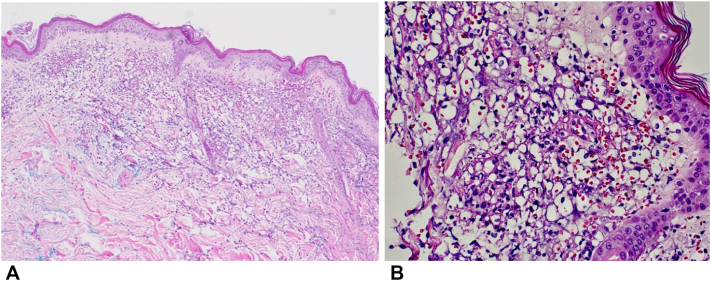
Acute bullous iododerma: focal thrombotic vasculopathy involving superficial dermal vascular channels, a focal subepidermal bulla, superficial dermal edema, and a mixed inflammatory infiltrate with scattered cryptococcoid bodies, **(A)** 10× and **(B)** 40× magnification. Hematoxylin and eosin stain.

A 62-year-old female with a history of stage 3 chronic kidney disease (intermittently on hemodialysis), type 2 diabetes mellitus, and hypertension presented to an outside hospital with facial droop after a fall. A CT head and CT angiogram of the chest with contrast was performed, and she was found to have a left middle cerebral artery M2 occlusion. Five days later she developed a bullous eruption that started under her left eye and spread rapidly to other areas of face, arms, anterior chest, and extremities. For evaluation of this new rash, she underwent CT scans of the left and right upper extremities with IV contrast the same day prior to transfer to our hospital, where she was evaluated by the dermatology service (Fig 5). Examination showed hemorrhagic bullae with marked predominance on the periorbital face, neck, upper chest, antecubital fossa, forearms, and hands (Fig 6). A punch biopsy of the arm showed small vessel vasculitis and thrombotic vasculopathy with scattered cryptococcoid bodies in the dermal inflammatory infiltrate. Direct immunofluorescence was negative. Other lab workup was remarkable for a negative cryoglobulin, antihistone IgG >7 U/mL (normal < 0.9 U/mL), p-ANCA >1:1280 (normal < 1:20), antimyeloperoxidase at 51 AU/mL (normal < 19 AU/mL), and anti-Scl70 at 2.4 U/mL (normal < 0.9 U/mL). Serum iodine levels collected 5 days after rash onset were markedly elevated at 47,964.2 μg/L (normal 40.0-92.0 μg/L). After treatment with IV and topical steroids as well as initiation of renal replacement therapy, the patient’s condition slowly improved.

## Discussion

Iododerma is a halogenoderma triggered by exposure to iodine-containing compounds such as iodinated contrast media, povidone-iodine, or amiodarone.^1^, ^2^, ^3^ Iododerma most commonly presents as an acneiform pustular eruption on the face and hands, while hemorrhagic bullae and secondary vasculitis are less frequently seen.^1^^,^^4^^,^^5^ In advanced stages, vegetative plaques may develop.^6^ These polymorphic clinical and histopathologic features often lead to delayed or incorrect diagnoses. The 3 cases presented here illustrate the less commonly seen bullous variant of iododerma.

Our 3 cases demonstrate features of CND—a histopathologic pattern in which a neutrophilic dermatosis displays features that mimic cutaneous cryptococcosis. This pattern was first reported in 2013 and has since been reported in a few patients with Sweet syndrome^7^^,^^8^ and only 4 patients with iododerma.^4^^,^^9^^,^^10^ Case 1 showed scant cryptococcoid bodies, case 3 showed scattered cryptococcoid bodies, and case 2 demonstrated extensive cryptococcoid bodies; this range ultimately highlights the spectrum of histopathologic findings that may not correlate with clinical severity.

The pathophysiology of cryptococcoid iododerma remains incompletely understood but is thought to involve iodine-induced neutrophil dysfunction and apoptosis.^11^ In patients with impaired renal clearance, iodine accumulation may exacerbate this effect. Hydralazine, a known trigger for neutrophilic dermatoses and ANCA-associated autoimmunity, may further potentiate neutrophil activation and injury.^12^ All 3 patients in our series had significant renal dysfunction and hydralazine exposure. All 3 cases also demonstrated elevated p-ANCA levels >1:1280, which is likely representative of the loss of self-tolerance and neutrophilic apoptosis induced by iododerma and potentiated by hydralazine. Notably, p-ANCA positivity can occur even without hydralazine exposure.^5^

Histopathology is important in differentiating iododerma from mimickers, and elevated urine and/or serum iodine levels can confirm the diagnosis. High serum iodine levels 2 days postexposure are considered pathologic.^13^ In our second and third case, iodine levels remained markedly elevated 10 and 5 days after exposure, respectively, nearly 200 and 600 times the upper limit of normal, respectively.

Sweet syndrome shares many features with iododerma, including abrupt onset, facial mucocutaneous, and upper extremity predilection and dense dermal neutrophilic infiltrates, including CND.^4^ This overlap makes it difficult to fully differentiate the 2 without further testing. However, symptomatically, Sweet syndrome nearly always presents with fever and systemic symptoms, while pyrexia is rare in reported cases of iododerma.^14^ Hydralazine-induced ANCA-associated vasculitis was also considered due to positive p-ANCA and antimyeloperoxidase antibodies. However, our patients did not exhibit clinical, histologic, or radiographic evidence of vasculitis. Evaluation for glomerulonephritis was limited by underlying renal impairment. Bullous lupus erythematosus was deemed unlikely based on morphology and nonspecific immunofluorescence studies. Ultimately, the temporal relationship between iodinated contrast exposure, clinicopathologic features, and elevated serum iodine levels (in cases 2 and 3), supported a unifying diagnosis of iododerma in all 3 patients.

Early recognition of acute bullous iododermas is critical, particularly in patients with chronic kidney disease/end stage renal disease or hydralazine use who develop a rapidly progressive, facial predominant mucocutaneous blistering eruption following iodine exposure. Histopathology may show CND. Treatment for iododerma typically involves avoiding further iodine exposure while trying to promote iodine excretion, such as dialysis, as well as systemic glucocorticoids to curtail the neutrophilic inflammatory response.^1^^,^^15^ Features that argue against alternative diagnoses include the absence of fever and lack of classic triggers for Sweet syndrome, and lack of evidence for multiorgan ANCA-associated vasculitis despite serologic positivity. Importantly, misdiagnosis of ANCA-associated vasculitis may lead to repeat contrast-enhanced imaging, which can exacerbate iododerma, as seen in case 1, resulting in clinical deterioration. Awareness of the clinical and histologic spectrum of iododerma, including CND, and the utility of serum iodine levels can aid in timely diagnosis and management of this rare condition.

## Conflicts of interest

None disclosed.
